# Mental health outcomes associated with electronic cigarette use, combustible tobacco use, and dual use among U.S. adolescents: Insights from the National Youth Tobacco Survey

**DOI:** 10.1371/journal.pmen.0000370

**Published:** 2025-07-23

**Authors:** Noor Abdulhay, R. Constance Wiener, Sijin Wen, Bethany Barone Gibbs, Ruchi Bhandari

**Affiliations:** 1 School of Public Health, West Virginia University, Morgantown, West Virginia, United States of America; 2 School of Dentistry, West Virginia University, Morgantown, West Virginia, United States of America; Manipal Academy of Higher Education, INDIA

## Abstract

Adolescence is a critical period for mental health development. There are increasing rates of anxiety, depression, and suicide among adolescents in the United States (U.S.). Tobacco use, especially electronic cigarettes (e-cigarettes) and dual use with combustible tobacco products (CTP), poses a significant threat to adolescent mental health. This study examines the association between various tobacco use profiles and mental health outcomes among U.S. middle and high school students. Data from the 2021–2023 National Youth Tobacco Survey were analyzed. Three-quarters of eligible students completed the survey across the three years. Depression and anxiety symptoms were assessed using the Patient Health Questionnaire (PHQ-4), with scores of ≥3 indicating probable cases, generating two binary variables (<3 vs. ≥ 3). Overall psychological distress was categorized into normal (0–2), mild (3–5), moderate (6–8), and severe (9–12) based on PHQ-4 total scores. Tobacco use was categorized as ever e-cigarette-only, ever CTP-only, ever dual use, and non-use. Descriptive statistics and weighted multivariable logistic and ordinal regression models estimated odds ratios (OR) and 95% confidence intervals (CI), adjusting for potential confounders. Among the 60,072 adolescents analyzed, 15,222 (25.21%) had depression and 17,790 (29.55%) had anxiety symptoms. Compared to adolescents who were non-users, those who were ever dual users had the highest odds, with adjusted OR of 1.90 (95%CI:1.58-1.94) for symptoms of depression, 1.58 (95%CI:1.40-1.78) for symptoms of anxiety, and 1.75 (95%CI:1.58-1.94) for overall psychological distress. Odds were also significantly higher for adolescents who exclusively used e-cigarettes compared to non-users and those who exclusively used CTP compared to non-users. This study highlights a significant association between tobacco use and poor mental health among adolescents. These results contribute to evidence informing future research and policy on adolescent tobacco use and mental health.

## Introduction

Adolescence is a critical window for the development of mental health. Longitudinal research has shown that youth with poor mental health are at a greater risk for adverse outcomes in adulthood [1]. Despite this concern, there has been little progress in addressing mental health concerns for this age group. For example, in 2023, 40% of high school students in the United States (U.S.) had reported experiencing feelings of sadness and hopelessness, a measure frequently used in national surveillance systems to screen for depressive symptoms [2]. Over the past decade, the percentage of high school students reporting feelings of sadness of hopelessness has increased among both male and female students [2]. Among female students, rates of feeling sad or hopeless rose from 39% in 2013 to 53% in 2023, while among male students, the rates increased from 21% to 28% during the same period [2].

In 2023, suicide was the second leading cause of death for youth aged 10 – 14 and those aged 15–24 [3]. In 2023, 20% of high school students seriously considered attempting suicide, 16% made a suicide plan, and 9% attempted suicide, rates that have all increased compared to 2013 [2]. Depression and anxiety are among the leading causes of illness and disability in adolescents, and both conditions are associated with suicidal behavior [4–6]. According to data from the National Health Interview Survey – Teen collected from July 2021 – December 2023, an estimated 17.8% of U.S. adolescents aged 12–17 reported symptoms of depression in the past two weeks, and 19.7% reported symptoms of anxiety [7].

Tobacco use and mental health challenges are known to have a complex, bidirectional relationship. A longitudinal study conducted in a large Midwestern city in the U.S. followed 262 girls aged 11–20 years old and found that higher levels of conventional cigarette smoking predicted increases in depressive symptoms over time, however, depressive symptoms did not predict smoking [8]. Another longitudinal study conducted in Southern California found that adolescents with higher depressive symptoms in 6^th^ grade were more likely to initiate smoking by 8^th^ grade [9]. Additionally, a study using data from the National Longitudinal Study of Adolescent to Adult Health (Add Health) identified a bidirectional association between conventional cigarette use and anxiety during adolescence [10]. This study found that adolescents who smoked more at baseline reported increased anxiety symptoms, and baseline anxiety symptoms also predicted greater cigarette use at follow up [10].

The rise of electronic cigarettes (e-cigarettes) has shifted tobacco consumption patterns, particularly among youth, who continue to be drawn to e-cigarettes for their perceived safety, discreet use, high nicotine content, and appealing flavors [11–14]. In 2024, approximately 5.28 million (19.0%) U.S. middle and high school students reported ever using any tobacco product, and 2.25 million (8.1%) students reported currently using any tobacco product [15]. E-cigarettes have remained the most used tobacco product since 2014, with 43.6% of ever e-cigarette users reporting current use [15,16]. The dual use of e-cigarettes and combustible tobacco products (CTP), such as conventional cigarettes, is also common, often leading to greater nicotine dependence and decreased motivation to quit [17–19].

Understanding the relationship between adolescent tobacco use and mental health in adolescents is particularly important as adolescence is a critical developmental period during which many health-related risk-taking behaviors begin [4]. Risk taking behaviors such as engaging in tobacco use are often unhealthy coping strategies for managing emotions and can negatively impact adolescent mental health [4]. Previous studies have proposed that mental health may be affected differently with e-cigarettes compared to CTP since e-cigarettes deliver nicotine without combustion, expose them to fewer harmful chemicals, and are used under different social and psychological contexts [20,21]. However, much of the current literature still focuses primary on conventional cigarettes, and there remains a significant gap in research examining how mental health is associated with e-cigarette use and dual use, particularly in comparison to conventional cigarette users or non- users [21].

Therefore, the aim of this study is to address this research gap by examining the association between different tobacco use profiles (e-cigarette, CTP, and dual use) and mental health outcomes (depression, anxiety, and overall psychological distress) among a nationally representative population of U.S. middle and high school students.

## Methods

### Study design and data source

This study is set within the most recent rounds of National Youth Tobacco Survey (NYTS), conducted between 2021–2023. The NYTS is a cross-sectional study that is conducted annually by the Centers for Disease Control and Prevention (CDC) [22]. This dataset includes comprehensive information on adolescents, such as demographic characteristics (age, gender, race/ethnicity), tobacco product use (types, frequency, and initiation), attitudes and beliefs towards tobacco use, exposure to tobacco marketing, and associated health behaviors [22]. The purpose of the data collection was to be able to support future tobacco control programs that can be implemented at the state and national level [22].

The inclusion criteria for the NYTS were that participants needed to be enrolled in either a public or private middle or high school in the U.S [23]. The NYTS uses a stratified three-stage cluster sample design including Primary Sampling Units (PSU), Secondary Sampling Units (SSU), and students from each selected school, to produce a nationally representative sample of middle and high school U.S. students [23]. PSU were defined as a county, or a group of small counties, or part of a very large county within each stratum and SSU were defined as schools within each selected PSU [23]. Once schools and specific classes were selected, the school coordinator would receive an email with instructions and parental permission forms, which needed to be distributed to students before the survey was conducted [23].

In 2021, the survey sampled 25,149 students, in 2022, it sampled 37,172 students, and in 2023, it sampled 31,108 students. The overall response rate across the three years was approximately 75.8%. A weighting factor was applied to each student record to ensure the weighted proportions of students in each grade match the national population proportions. Full details of the NYTS methodology, including information about survey weights, are available elsewhere [23].

### Ethics statement

This study used publicly available, de-identified data from the NYTS, collected by the CDC. All analyses were conducted in accordance with CDC’s standards for the NYTS data use, which prohibit any attempt to identify individuals or schools [22]. The data are publicly accessible and do not contain any personally identifiable information [23].

### Measures

#### Depression, anxiety, and overall psychological distress.

The dependent variables, self-reported symptoms of depression, anxiety, and overall psychological distress, were assessed using the Patient Health Questionnaire–4 (PHQ-4). The PHQ-4 is a brief, self-administered screening tool used to identify symptoms of depression, anxiety, and overall psychological distress. Internal reliability and validity for the PHQ-4 is well established in previous studies [24,25].

Participants are asked to rate how frequently they experienced four different symptoms on a scale of “not at all”, “several days”, more than half the days”, or “nearly every day”. The first two symptoms are specific to depression (“little interest or pleasure in doing things” and “feeling down, depressed, or hopeless”) and the third and fourth symptoms are specific to anxiety (“feeling nervous, anxious, or on edge” and “not being able to stop or control worrying”). Each response option is assigned a score of 0–3 with higher scores indicating greater severity of symptoms.

Total scores of 3 or higher for the first two questions suggest probable depression and were coded as ‘depression’ in the current analysis [25]. Total scores of 3 or higher for the last two questions suggest probable anxiety and were coded as ‘anxiety’ for the current analysis [25]. Both depression and anxiety were coded as binary variables. Overall psychological distress was calculated by summing the scores for all four questions, with scores of 0–2 considered normal distress, scores of 3–5 considered mild distress, scores of 6–8 considered moderate distress, and scores of 9–12 considered severe distress [25]. Overall psychological distress was coded as an ordinal variable.

#### Tobacco use categories.

The primary independent variable, tobacco smoking category, was derived from several questions from the NYTS. Adolescents who exclusively used e-cigarettes were labeled as having “ever e-cigarette-only use”. To be categorized as such, participants must have answered “yes” to the question, “Have you ever used an electronic cigarette, even once or twice?”. Additionally, they must have responded “no” to any questions regarding the use of CTP. Adolescents who exclusively used CTP products were labeled as having “ever CTP-only use”. To be categorized as such, participants must have answered “yes” to questions about cigarette smoking, cigar smoking, hookah or waterpipe use, tobacco-filled pipes smoking, or bidis smoking, while also answering “no” to the question about e-cigarette use. Adolescents who used both e-cigarettes and CTP were categorized as having “ever dual use”. Dual use was identified by an answer of “yes” to questions related to both e-cigarette and CTP use, indicating usage of both products. Finally, adolescents who did not use tobacco were categorized as having “non-use”. Non-use was identified by an answer of “no” to questions related to both e-cigarette and CTP use, indicating lack of usage by either product.

#### Control variables.

Potential confounders measured on the self-report survey included level of school (middle school/high school), sex (male/female), race/ethnicity (White/Black/Hispanic/Asian/other), sexual orientation (heterosexual/ gay, lesbian, or bisexual/not sure), tobacco use in household (yes/no), social media usage (never/few times a week/1–2 hours a day/ ≥ 3 hours a day), and average school grades during the past 12 months (mostly A-B/mostly C-D/mostly F/no grade or not sure).

### Statistical analysis

Descriptive statistics of all participant characteristics are presented as unweighted frequencies and weighted percentages. Rao-Scott Chi-square tests were used to compare participant characteristics among tobacco smoking categories.

Three separate weighted regression models were used to examine the association between the tobacco smoking categories and symptoms of depression, anxiety, and overall psychological distress. All regression analyses incorporated the complex sampling design of the NYTS, accounting for sampling weights, clusters, and stratification variables. For each regression model, unadjusted odds ratio (OR) and adjusted odds ratio (aOR) with 95% confidence intervals (CI) are reported.

In the first and second models, weighted multivariable logistic regression was used with symptoms of depression and anxiety as the main outcome variables, respectively. In the third model, a weighted ordinal logistic regression was used with overall psychological distress as the main outcome variable.

Model fit for the logistic regression analyses was assessed using the concordance statistic, and all model assumptions were met. Complete case analysis was carried out if there were missing observation in the used variables. Participants were excluded from the study if they did not complete the mental health PHQ-4 questionnaire or if any tobacco-related questions were unanswered. Significance level was set to P ≤ .05. All statistical analysis were conducted using R version 4.1.1.

## Results

**Fig 1:**
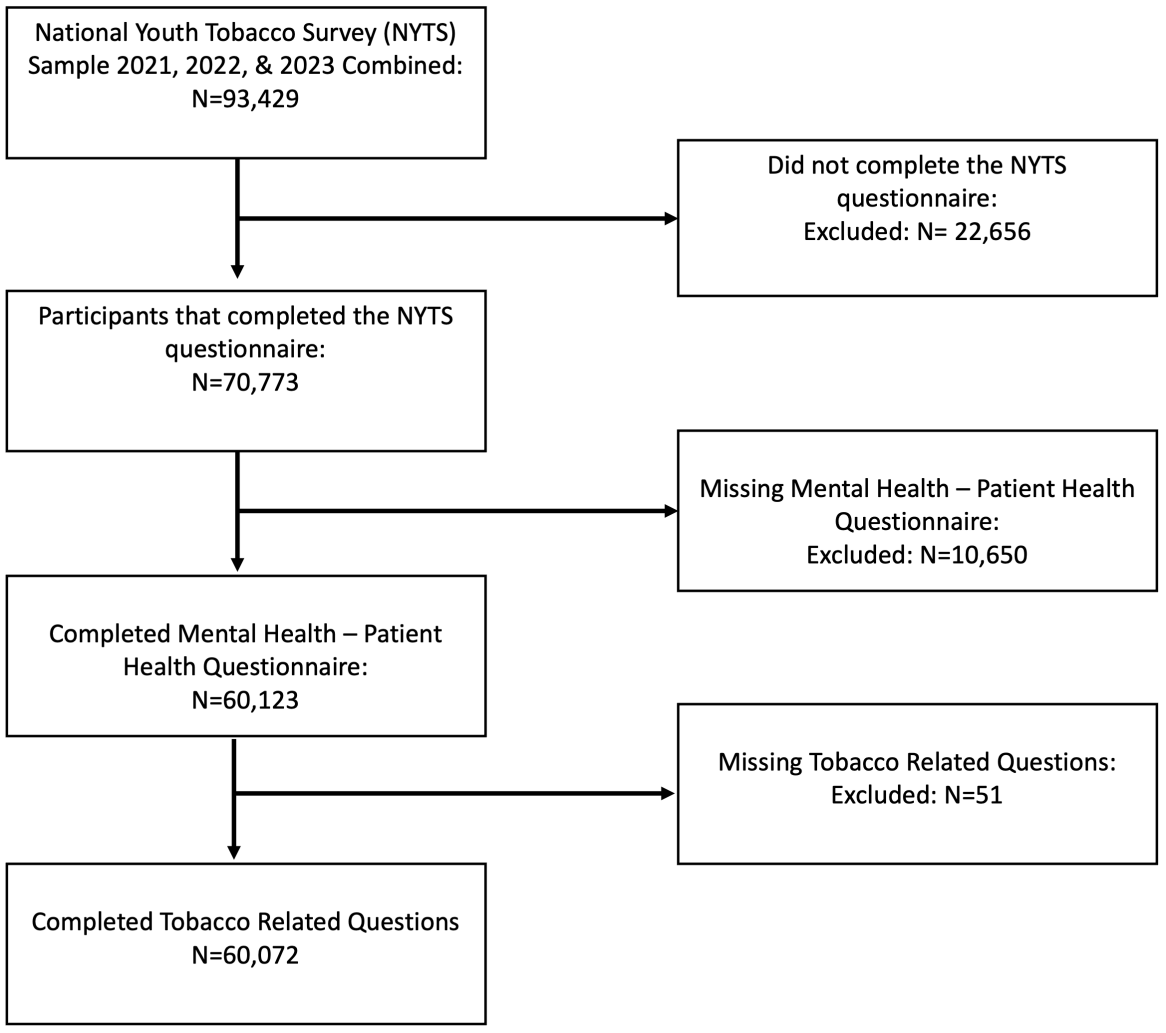
Flow Diagram of Participant Selection from the National Youth Tobacco Survey (NYTS). The diagram illustrates the selection process of participants included in the analysis from the combined 2021, 2022, and 2023 NYTS samples.

Of the 93,429 middle and high school students initially included in the NYTS sample from 2021-2023, 70,773 completed the student questionnaires. After further excluding 10,701 participants who did not complete the PHQ-4 or had incomplete answers on tobacco use-related questions, a total of 60,072 (62.4%) participants were included in the analysis (Fig 1). A comparison of participants who were included in the analysis (n = 60,072) versus those who were excluded (n = 10,701) is provided in S2 Table. Participants who were excluded were more likely to be part of the 2023 cycle, in middle school, male, Black or Hispanic, unsure about their sexual orientation, from households with tobacco use, using social medial less frequency, and achieving lower grades, all with P < 0.001. However, given the large sample size, the magnitude of difference between excluded and included participants were minimal.

Descriptive statistics across the tobacco smoking categories are presented in Table 1. Among the surveyed participants, there were 2,306 who had ever CTP-only use (3.61%), 5,686 had ever e-cigarette-only use (9.94%), 4,605 had ever dual use (7.80%), and 47,475 who had no use (78.63%). Significant differences in participant characteristics across tobacco smoking categories were observed in school level, sex, race/ethnicity, sexual orientation, tobacco use in the household, social media usage, and average school grades, all with p-values < .001. Compared to middle school students, high school students had higher rates of ever e-cigarette only use (4.6% vs. 13.9%) and ever dual use (3.1% vs. 11.2%). Compared to heterosexual participants, sexual minority youth had a higher prevalence of ever e-cigarette only use (9.9% vs. 13.1%) and ever dual use (7.5% vs. 14.1%). Social media usage also correlated with tobacco use. Compared to participants who never used social media, those who used social media for 3 or more hours per day reported great use for ever e-cigarette use (9.9% vs. 12.1%) and ever dual use (7.5% vs. 12.1%). Finally, adolescents with lower academic performance had higher rates of tobacco use, with ever dual use most common among those with mostly F’s (19.2%) compared to those with mostly A-B’s (6.5%).

**Table 1:** Participant Charactertiscs Among Tobacco Use Status.

		*Smoking Status*				
Variables:	Total N (%)	Ever CTP-Only Use N (%)^a^	Ever E-cigarette -Only Use N (%)^a^	Ever Dual Use N (%)^a^	Non-Use N (%)^a^	P-Value^b^
Total N (%)		2,306 (3.61%)	5,686 (9.94%)	4,605 (7.80%)	47,475 (78.63%)	
***Year***						**<.001**
2021	17,336 (33.41%)	685 (3.68%)	1,724 (10.8%)	1,293 (7.80%)	13,634 (77.7%)	
2022	24,236 (33.28%)	961 (3.63%)	2,575 (10.6%)	2,153 (8.87%)	18,547 (76.9%)	
2023	18,500 (33.30%)	660 (3.55%)	1,387 (8.41%)	1,159 (6.74%)	15,294 (81.3%)	
***School Type***						**<.001**
Middle School	27,560 (49.21%)	874 (3.04%)	1,307 (4.62%)	968 (3.10%)	24,411 (89.2%)	
High School	32,377 (50.37%)	1,420 (4.01%)	4,375 (13.9%)	3,620 (11.2%)	22,962 (70.9%)	
***Sex***						**<.001**
Male	29,981 (50.37%)	1,204 (3.23%)	2,486 (11.4%)	2,229 (7.98%)	24,062 (77.4%)	
Female	29,820 (49.21%)	1,095 (4.01%)	3,181 (8.53%)	2,353 (7.63%)	23,191 (79.8%)	
***Race/Ethnicity***						**<.001**
White	29,254 (53.19%)	879 (3.06%)	3,147 (11.3%)	2,530 (9.08%)	22,698 (76.5%)	
Black	7,769 (12.33%)	561 (6.57%)	509 (6.66%)	468 (6.01%)	6,231 (80.8%)	
Hispanic	15,360 (25.41%)	581 (3.70%)	1,534 (9.91%)	1,186 (7.34%)	12,059 (79.1%)	
Asian	4,405 (5.91%)	88 (2.09%)	223 (6.30%)	110 (2.65%)	3,984 (89.0%)	
Other	2,137 (1.25%)	150 (4.73%)	216 (8.63%)	256 (8.57%)	1,515 (78.1%)	
***Sexual Orientation***						**<.001**
Heterosexual	41,211 (69.07%)	1,491 (3.34%)	3,992 (9.94%)	2,972 (7.47%)	32,756 (79.2%)	
Sexual Minority	8,531 (14.28%)	471 (5.72%)	1,101 (13.1%)	1,183 (14.1%)	5,776 (67.1%)	
Not sure	8,142 (13.37%)	288 (3.04%)	489 (7.00%)	373 (3.85%)	6,992 (86.1%)	
***Tobacco Use in Household***						
Yes	17,707 (29.32%)	1,039 (5.43%)	2,430 (13.8%)	2,506 (14.2%)	11,732 (66.5%)	
No	40,541 (67.80%)	1,190 (2.81%)	3,067 (8.24%)	1,955 (5.00%)	34,329 (84.0%)	
***Social Media Usage***						**<.001**
Never	5211 (8.31%)	172 (2.95%)	224 (4.66%)	212 (4.11%)	4,603 (88.3%)	
Few times a week	5,820 (9.29%)	240 (4.96%)	360 (5.79%)	382 (5.79%)	4,838 (82.2%)	
1–2 hours a day	15,443 (25.75%)	462 (2.84%)	1,099 (7.88%)	935 (6.36%)	12,947 (82.9%)	
3 + hours a day	33,327 (56.20%)	1,420 (3.86%)	3,967 (12.1%)	3,043 (9.20%)	24,897 (74.7%)	
***Average Grades***						**<.001**
Mostly A-Bs	44,266 (73.49%)	1,399 (3.09%)	3,944 (9.52%)	2,736 (6.52%)	36,187 (80.9%)	
Mosty C-Ds	9,531 (15.81%)	545 (4.97%)	1,222 (12.5%)	1,264 (12.5%)	6,500 (70.9%)	
Mostly Fs	1,336 (2.43%)	109 (8.20%)	188 (14.0%)	254 (19.2%)	785 (58.6%)	
No Grade/Not sure	4,508 (7.58%)	236 (4.23%)	297 (7.14%)	313 (6.81%)	3,662 (81.8%)	

^a^Weighted population estimates.

^b^P-Value for Rao-Scott Chi-square test.

Note: Boldface indicates statistical significance.

**Fig 2:**
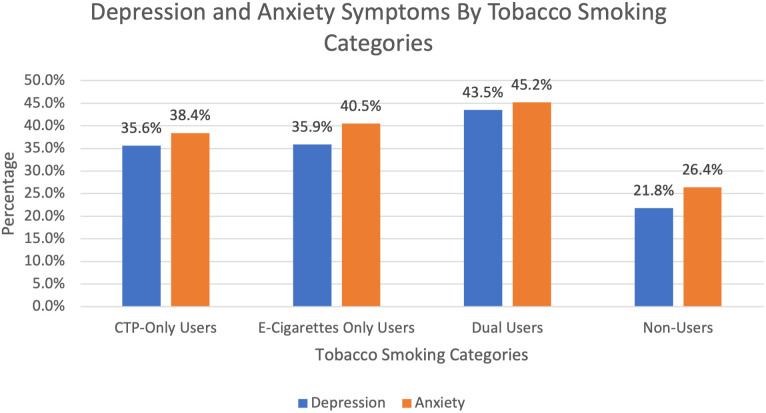
Depression and Anxiety Symptoms by Tobacco Smoking Categories. The figure illustrates the percentage of participants reporting depression and anxiety symptoms within four tobacco smoking categories. Combustible Tobacco Products (CTP) only, electronic cigarette (e-cigarette) only, dual use (CTP and e-cigarettes), and non-use.

The prevalence of depression and anxiety symptoms across different tobacco smoking categories is presented in Fig 2. Among adolescents who were ever dual users, 43.4% reported symptoms of depression and 45.2% reported symptoms of anxiety. For adolescents who were ever e-cigarette-only users, 35.9% reported symptoms of depression and 40.5% indicated symptoms of anxiety. Adolescents who ever used CTP-only reported similar rates (35.6% for depression, 38.4% anxiety). Adolescents who did not use any tobacco reported the lowest prevalence, with 21.8% reporting depression symptoms, and 26.4% reporting anxiety symptoms.

Unadjusted OR and aOR for the association between tobacco smoking categories and symptoms of depression, anxiety, and overall psychological distress are presented in Table 2. The adjusted analysis revealed that compared to adolescents with no use, adolescents with ever CTP-only use had increased odds of depression (aOR = 1.52, 95% CI: 1.29-1.78), anxiety (aOR = 1.39, 95% CI: 1.18-1.63), and psychological distress (aOR = 1.43, 95% CI: 1.24-1.65). Adolescents with ever e-cigarette-only use had even higher odds of depression (aOR = 1.67, 95% CI: 1.50-1.85), anxiety (aOR = 1.48, 95% CI: 1.34-1.65), and psychological distress (aOR = 1.63, 95% CI: 1.49-1.78). Finally, adolescents with ever dual use exhibited the greatest risk, with aORs of 1.90 (95% CI: 1.69-2.14) for depression, 1.58 (95% CI: 1.40-1.78) for anxiety, and 1.75 (95% CI: 1.58-1.94) for psychological distress (Table 2). Across all three mental health outcomes, the aORs were slightly lower compared to the unadjusted estimates, indicating that part of the association was explained by covariates. However, all associations remained statistically significant after adjustment. The full models, including associations with demographic factors, are reported in the supplementary material (S3, S4, S5 Tables).

**Table 2:** Unadjusted and Adjusted Odds Ratios for Association between Tobacco Use Categories and Mental Health Outcomes.

	Depression - Unadjusted OR (95% CI)	Depression - Adjusted OR (95% CI)	Anxiety - Unadjusted OR (95% CI)	Anxiety - Adjusted OR (95% CI)	Psychological Distress - Unadjusted OR (95% CI)	Psychological Distress - Adjusted OR (95% CI)
Tobacco Use Status						
Non-Use	1	1	1	1	1	1
Ever CTP-Only Use	**1.87 (1.62, 2.16)**	**1.52 (1.29,1.78)**	**1.61 (1.40, 1.86)**	**1.39 (1.18, 1.63)**	**1.70 (1.49, 1.94)**	**1.43 (1.24, 1.65)**
Ever E-cigarette-Only Use	**2.06 (1.86,2.29)**	**1.67 (1.50,1.85)**	**1.91 (1.73, 2.11)**	**1.48 (1.34, 1.65)**	**2.13 (1.94, 2.35)**	**1.63 (1.49, 1.78)**
Ever Dual Use	**2.58 (2.34,2.86)**	**1.90 (1.69, 2.14)**	**2.19 (1.98, 2.45)**	**1.58 (1.40, 1.78)**	**2.48 (2.27, 2.71)**	**1.75 (1.58, 1.94)**

Note: Adjusted models controlled for potential confounding factors including school level, sex, race/ethnicity, tobacco use in the household, social media usage, and school grades. Boldface indicates statistical significance.

## Discussion

Results from this study highlight how tobacco use, particularly ever e-cigarette-only use and ever dual use, is associated with mental health conditions among adolescents. Our results demonstrate that, compared to adolescents who have never used any tobacco product, adolescents who have ever used e-cigarettes, CTP, and especially both, had higher odds of experiencing symptoms of depression, anxiety, and overall psychological distress. These associations remained statistically significant even after adjusting for demographic and behavioral covariates.

Results from this study are consistent with the limited studies that have explored the relationship between tobacco use, including e-cigarette use, and mental health. For example, a study using data from the 2019 Texas Adolescent Tobacco and Marketing Surveillance study found that youth with negative mental health had significantly higher odds of using tobacco products, including e-cigarettes [26]. Similarly, a study conducted among Thai adolescents found that those who had ever used e-cigarettes were more likely to show signs of depression, even if they were not currently using that product [27]. A previous longitudinal study among high school students in Los Angeles, California examined the bidirectional association between tobacco use, including e-cigarettes and dual use, and depressive symptoms [20]. This study found that higher depressive symptoms at baseline significantly increased the odds of initiating tobacco use at follow up [20]. Additionally, e-cigarette use over time was associated with a greater increase in depressive symptoms, however this was not statistically significant [20].

In addition to just experiencing poor mental health, e-cigarette use has also been associated with suicidal behaviors among youth. A study using data from the 2017 and the 2019 Youth Risk Behavior Survey in the U.S. revealed that current users of electronic vaping products had significantly greater odds of suicidal ideation, suicide plans, and suicide attempts compared to never users [28]. Additionally, a nationally representative study of Korean adolescents further showed that e-cigarette and dual users were both about 6 times more likely to have attempted suicide and conventional cigarette only users were around 2 times more likely to attempt suicide than non-users [29].

The observed associations between different tobacco use products and mental health symptoms may be explained by several mechanisms. Both e-cigarettes and CTP differ in their delivery mechanisms, however, both methods expose adolescents to nicotine, a highly addictive substance [30]. Adolescents who develop a dependency to nicotine, may experience disruptions in brain development, potentially affecting mood regulation, permanent damage to memory, cognitive function, and emotional control [31–34].

### Strengths and limitations

Our study has several strengths. Our results present the most recent data concerning adolescents and their relationship with tobacco use products and mental health. The rising concerns about mental health among adolescents make this research especially relevant in the current public health landscape. Data for this study were obtained from a large nationally representative sample of U.S. adolescent population, providing excellent external validity. Another notable strength of this study is that the mental health conditions were reported directly by the adolescents themselves, rather than relying on parent of guardian reports, which is often the case in mental health studies for adolescents. While prior research has primarily concentrated on conventional cigarette use, this study expands the scope by comparing mental health outcomes across distinct tobacco use profiles (exclusive e-cigarette use, exclusive CTP use, and dual use).

However, there are limitations to this study. The study's cross-sectional design precludes the establishment of causality between tobacco use and mental health outcomes among adolescents. The reliance on self-reported data may have introduced bias. Additionally, recall bias could have influenced the accuracy of reported information. However, since this study was restricted to adolescents only, the recall bias may not be significant. Additionally, the NYTS had limited variables on other health-related factors that may have confounded associations between adolescents mental health and tobacco use. Inclusion of covariate adjustment for behavioral factors such as diet, exercise, and sleep patterns, as well as socioeconomic status, could improve future studies. While the NYTS has good generalizability to the adolescent population in the U.S., this survey only included students who attended public and private schools, thereby excluding adolescents who are not in traditional school settings. Finally, the sample size for tobacco use available in NYTS did not allow for the examination of the dose-response relationship between tobacco use and mental health. Such analyses will be important for future studies to determine risk gradients and better establish causal relationships.

### Implications for future research

Future longitudinal research should explore the bidirectional nature of the relationship between tobacco use and mental health outcomes among adolescents, with a focus on e-cigarette and dual use which have not been widely studied in this population. This research should examine the temporal sequence of events, determining whether poor mental health precedes tobacco use, or whether tobacco use exacerbates pre-existing mental health issues, or both. Additionally, future research should investigate the long-term trends and behaviors associated with using multiple tobacco products among adolescents over an extended period. To further clarify these complex relationships, future studies could also consider alternative analytical approaches, such as Structural Equation Modeling, to explore directionality of effects in conjunction with casual theory.

### Implications for policy and practice

Results from this study have direct implications for public health policy and interventions aimed at addressing the adolescent mental health crisis. Given the current adolescent mental health crisis, addressing tobacco use, especially e-cigarettes, may be a critical component in mitigating these mental health challenges. Although recent data suggest tobacco use among adolescents is declining, these products remain highly popular [35], and their association with mental health problems continue to be a public health concern. Therefore, it is essential for healthcare providers, parents, and educators to be aware of the heightened mental health risks associated with e-cigarette use. Clinicians and health professionals should be encouraged to screen for mental health issues in adolescents who report tobacco use, and likewise, assess for substance use among youth being seen for mental health issues. Furthermore, public health campaigns and educational efforts must emphasize the mental health risks associated with e-cigarette, conventional cigarette, and dual use, to provide adolescents and the broader public with a comprehensive understanding of the risks posed by these products. Stronger regulations on the sale and advertisements of e-cigarettes and CTP targeting young people should be considered. By integrating the findings from this study into policy, practice, and public awareness, we can better address the dual challenges of tobacco use and mental health in adolescents in the U.S.

## Conclusion

In a nationally representative sample of middle and high school U.S. students, adolescents who used tobacco were more likely to report negative mental health conditions compared to non-users. While causality cannot be determined, the results from this study showed that all forms of tobacco use were significantly associated with mental health issues. There is a need to continue promoting mental health support and implementing tailored interventions to combat all forms of tobacco use among adolescents.

## Supporting information

S1 Table This table presents the distribution of the variables among the participants. Missing values are reported.
(DOCX)

S2 Table This table compares the characteristics of participants included in the sample (n = 60,072) with those who were excluded due to missing data (n = 10,701). (DOCX)

S3 Table This table shows the odds ratio and 95% confidence intervals from both unadjusted and adjusted regression models between the independent variables and depression. (DOCX)

S4 Table This table shows the odds ratio and 95% confidence intervals from both unadjusted and adjusted regression models between the independent variables and anxiety. (DOCX)

S5 Table This table shows the odds ratio and 95% confidence intervals from both unadjusted and adjusted regression models between the independent variables and psychological distress. (DOCX)

## References

[1] Copeland WE, Wolke D, Shanahan L, Costello EJ. Adult functional outcomes of common childhood psychiatric problems: a prospective, longitudinal study. JAMA Psychiatry. 2015;72(9):892–9. 26176785 10.1001/jamapsychiatry.2015.0730 PMC4706225

[2] Centers for Disease Control and Prevention (CDC). Youth Risk Behavior Survey (YRBS) Data Summary & Trends Report. Atlanta: CDC; 2024. Available from: https://www.cdc.gov/yrbs/dstr/index.html

[3] Centers for Disease Control and Prevention (CDC). WISQARS Leading Causes of Death Reports, 2023. Atlanta: CDC; 2024. Available from: https://wisqars.cdc.gov/lcd/?o=LCD&y1=2023&y2=2023&ct=10&cc=ALL&g=00&s=0&r=0&ry=2&e=0&ar=lcd1age&at=groups&ag=lcd1age&a1=0&a2=199

[4] World Health Organization (WHO). Adolescent mental health. Geneva: WHO; 2021. Available from: https://www.who.int/news-room/fact-sheets/detail/adolescent-mental-health

[5] Fang L, Tong Y, Li M, Wang C, Li Y, Yuan M, et al. Anxiety in adolescents and subsequent risk of suicidal behavior: A systematic review and meta-analysis. J Affect Disord. 2024. 10.1016/j.jad.2024.05.005 38703913

[6] Grossberg A, Rice T. Depression and Suicidal Behavior in Adolescents. Med Clin North Am. 2023;107(1):169–82. doi: 10.1016/j.mcna.2022.04.005 36402497

[7] Centers for Disease Control and Prevention (CDC). National Health Interview Survey (NHIS) Teen Data Query Tool. 2024. Available from: https://wwwn.cdc.gov/NHISDataQueryTool/NHIS_TEEN/index.html

[8] Beal SJ, Negriff S, Dorn LD, Pabst S, Schulenberg J. Longitudinal associations between smoking and depressive symptoms among adolescent girls. Prev Sci. 2014;15(4):506–15. doi: 10.1007/s11121-013-0402-x 23689842 PMC3800222

[9] Weiss JW, Mouttapa M, Cen S, Johnson CA, Unger J. Longitudinal effects of hostility, depression, and bullying on adolescent smoking initiation. J Adolesc Health. 2011;48(6):591–6. 21575819 10.1016/j.jadohealth.2010.09.012 PMC3096829

[10] Bilsky SA, Luber MJ, Cloutier RM, Dietch JR, Taylor DJ, Friedman HP. Cigarette use, anxiety, and insomnia from adolescence to early adulthood: A longitudinal indirect effects test. Addict Behav. 2021;120:106981. 33993036 10.1016/j.addbeh.2021.106981 PMC12162129

[11] Amrock SM, Lee L, Weitzman M. Perceptions of e-cigarettes and noncigarette tobacco products among US youth. Pediatrics. 2016;138(5). 10.1542/peds.2015-4306 PMC5079074 27940754

[12] Ramamurthi D, Chau C, Jackler RK. JUUL and other stealth vaporisers: hiding the habit from parents and teachers. Tob Control. 2019;28(6):610–6. 10.1136/tobaccocontrol-2018-054455 30219794

[13] Goniewicz ML, Boykan R, Messina CR, Eliscu A, Tolentino J. High exposure to nicotine among adolescents who use Juul and other vape pod systems (‘pods’). Tob Control. 2019;28(6):676–7. 30194085 10.1136/tobaccocontrol-2018-054565 PMC6453732

[14] Pepper JK, Ribisl KM, Brewer NT. Adolescents’ interest in trying flavoured e-cigarettes. Tob Control. 2016;25(Suppl 2):ii62–6. 10.1136/tobaccocontrol-2016-053174 PMC5125087 27633762

[15] Jamal A. Tobacco product use among middle and high school students—National Youth Tobacco Survey, United States, 2024. MMWR Morb Mortal Wkly Rep. 2024;73. 10.15585/mmwr.mm7341a2 PMC11486349 39418216

[16] Park-Lee E, Ren C, Cooper M, Cornelius M, Jamal A, Cullen KA. Tobacco product use among middle and high school students — United States, 2023. MMWR Morb Mortal Wkly Rep. 2023;73(41):869–76. 10.15585/mmwr.mm7145a1 PMC9707354 36355596

[17] Centers for Disease Control and Prevention (CDC). Youth and tobacco use. Centers for Disease Control and Prevention. 2024; Available from: https://www.cdc.gov/tobacco/php/data-statistics/youth-data-tobacco/index.html

[18] Wang TW. Tobacco product use and associated factors among middle and high school students—United States, 2019. MMWR Surveill Summ. 2019;68. 10.15585/mmwr.ss6812a1 PMC6903396 31805035

[19] Strong DR, Pearson J, Ehlke S, Kirchner T, Abrams D, Taylor K, et al. Indicators of dependence for different types of tobacco product users: Descriptive findings from Wave 1 (2013–2014) of the Population Assessment of Tobacco and Health (PATH) study. Drug Alcohol Depend. 2017;178:257–66. 28675817 10.1016/j.drugalcdep.2017.05.010

[20] Manzoli L, Flacco ME, Ferrante M, La Vecchia C, Siliquini R, Ricciardi W, et al. Cohort study of electronic cigarette use: effectiveness and safety at 24 months. Tob Control. 2017;26(3):284–92. 27272748 10.1136/tobaccocontrol-2015-052822 PMC5520273

[21] Lechner WV, Janssen T, Kahler CW, Audrain-McGovern J, Leventhal AM. Bi-directional associations of electronic and combustible cigarette use onset patterns with depressive symptoms in adolescents. Prev Med. 2017;96:73–8. 28024859 10.1016/j.ypmed.2016.12.034 PMC5510594

[22] Leventhal AM, Strong DR, Sussman S, Kirkpatrick MG, Unger JB, Barrington-Trimis JL, et al. Psychiatric comorbidity in adolescent electronic and conventional cigarette use. J Psychiatr Res. 2016;73:71–8. 26688438 10.1016/j.jpsychires.2015.11.008 PMC4738156

[23] Centers for Disease Control and Prevention. National Youth Tobacco Survey (NYTS). CDC; 2024. Available from: https://www.cdc.gov/tobacco/about-data/surveys/national-youth-tobacco-survey.html

[24] Centers for Disease Control and Prevention. Historical NYTS Data and Documentation. CDC; 2024. Available from: https://www.cdc.gov/tobacco/about-data/surveys/historical-nyts-data-and-documentation.html

[25] Materu J, Kuringe E, Nyato D, Galishi A, Mwanamsangu A, Katebalila M, et al. The psychometric properties of PHQ-4 anxiety and depression screening scale among out of school adolescent girls and young women in Tanzania: a cross-sectional study. BMC Psychiatry. 2020;20(1):321. doi: 10.1186/s12888-020-02735-5 32560705 PMC7304148

[26] Kroenke K, Spitzer RL, Williams JBW, Löwe B. An ultra-brief screening scale for anxiety and depression: the PHQ-4. Psychosomatics. 2009;50(6):613–21. doi: 10.1176/appi.psy.50.6.613 19996233

[27] Sumbe A, Wilkinson AV, Clendennen SL, Bataineh BS, Sterling KL, Chen B, et al. Association of tobacco and marijuana use with symptoms of depression and anxiety among adolescents and young adults in Texas. Tob Prev Cessat. 2022;8:3. 10.18332/tpc/144500 PMC8792993 35128214

[28] Patanavanich R, Vityananan P, Neelapaichit N, Chariyalertsak S, Kessomboon P, Assanangkornchai S, et al. Association between electronic cigarette use and depression among Thai adolescents: The Thailand National Health Examination Survey 2019–2020. Tob Induc Dis. 2022;20:103. 36447457 10.18332/tid/155333 PMC9673242

[29] Baiden P, Szlyk HS, Cavazos-Rehg P, Onyeaka HK, Peoples JE, Kasson E. Use of electronic vaping products and mental health among adolescent high school students in the United States: The moderating effect of sex. J Psychiatr Res. 2022;147:24–33. 35007808 10.1016/j.jpsychires.2021.12.050 PMC8905685

[30] Kim JS, Kim K. Electronic cigarette use and suicidal behaviors among adolescents. J Public Health (Oxf). 2021;43(2):274–80. doi: 10.1093/pubmed/fdz086 31334765

[31] Fadus MC, Smith TT, Squeglia LM. The rise of e-cigarettes, pod mod devices, and JUUL among youth: Factors influencing use, health implications, and downstream effects. Drug Alcohol Depend. 2019;201:85–93. 31200279 10.1016/j.drugalcdep.2019.04.011 PMC7183384

[32] Benowitz NL. Nicotine addiction. N Engl J Med. 2010;362(24):2295–303. 20554984 10.1056/NEJMra0809890 PMC2928221

[33] DiFranza JR, Wellman RJ, Mermelstein R, Pbert L, Klein JD, Sargent JD. The natural history and diagnosis of nicotine addiction. Curr Pediatr Rev. 2011;7(2):88–96. 10.2174/1573396311666150501002703 25938380

[34] Leslie FM. Unique, long-term effects of nicotine on adolescent brain. Pharmacol Biochem Behav. 2020;197:173010. 32738256 10.1016/j.pbb.2020.173010 PMC7484459

[35] U.S. Food and Drug Administration. Youth e-cigarette use drops to lowest level in a decade. FDA Newsroom. 2024. Available from: https://www.fda.gov/news-events/press-announcements/youth-e-cigarette-use-drops-lowest-level-decade

