# Supplementary material for: Mental health outcomes associated with electronic cigarette use, combustible tobacco use, and dual use among U.S. adolescents: Insights from the National Youth Tobacco Survey
Source: PLOS Ment Health. 2025 Jul 23;2(7):e0000370. doi: 10.1371/journal.pmen.0000370 (PMC12798231; doi:10.1371/journal.pmen.0000370)
Supplement: S1 Table — Missing values are reported. (DOCX) [file pmen.0000370.s001.docx]

| **S1 Table:** Participant Characteristics Including Missing Data | |  |
| --- | --- | --- |
| Variables: | **Total N (%)**  60,072 |  |
| ***Year*** |  |  |
| 2021 | 17,336 (33.41%) |  |
| 2022 | 24,236 (33.28%) |  |
| 2023 | 18,500 (33.30%) |  |
| ***School Type*** |  |  |
| Middle School | 27,560 (49.21%) |  |
| High School | 32,377 (50.37%) |  |
| Missing | 135 (0.22%) |  |
| ***Sex*** |  |  |
| Male | 29,981 (50.37%) |  |
| Female | 29,820 (49.21%) |  |
| Missing | 271 (0.43%) |  |
| ***Race/Ethnicity*** |  |  |
| White | 29,254 (53.19%) |  |
| Black | 7,769 (12.33%) |  |
| Hispanic | 15,360 (25.41%) |  |
| Asian | 4,405 (5.91%) |  |
| Other | 2,137 (1.25%) |  |
| Missing | 1,147 (1.9%) |  |
| ***Sexual Orientation*** |  |  |
| Heterosexual | 41,211 (69.07%) |  |
| Sexual Minority | 8,531 (14.28%) |  |
| Not sure | 8,142 (13.37%) |  |
| Missing | 2,188 (3.6%) |  |
| ***Tobacco Use in Household*** |  |  |
| Yes | 17,707 (29.32%) |  |
| No | 40,541 (67.80%) |  |
| Missing | 1,824 (2.90%) |  |
| ***Social Media Usage*** |  |  |
| Never | 5,211 (8.31%) |  |
| Few times a week | 5,820 (9.29%) |  |
| 1-2 hours a day | 15,443 (25.75%) |  |
| 3+ hours a day | 33,327 (56.20%) |  |
| Missing | 271 (0.43%) |  |
| ***Average Grades*** |  |  |
| Mostly A-Bs | 44,266 (73.49%) |  |
| Mosty C-Ds | 9,531 (15.81%) |  |
| Mostly Fs | 1,336 (2.43%) |  |
| No Grade/Not sure | 4,508 (7.58%) |  |
| Missing | 431 (0.70%) |  |
| ^a^ Weighted population estimates.  ^b^ P-Value for Rao-Scott Chi-square test  Note: Boldface indicates statistical significance | | |
