# Supplementary material for: Mental health outcomes associated with electronic cigarette use, combustible tobacco use, and dual use among U.S. adolescents: Insights from the National Youth Tobacco Survey
Source: PLOS Ment Health. 2025 Jul 23;2(7):e0000370. doi: 10.1371/journal.pmen.0000370 (PMC12798231; doi:10.1371/journal.pmen.0000370)
Supplement: S2 Table — (DOCX) [file pmen.0000370.s002.docx]

| **S2 Table:** Participant Characteristic Comparison of Included vs. Excluded | | | |
| --- | --- | --- | --- |
| **Characteristic** | **Included (n = 60,072)** | **Excluded (n = 10,701)** | **P-Value** |
| ***Year*** |  |  | **<.001** |
| 2021 | 33.41% | 31.63% |  |
| 2022 | 33.28% | 31.19% |  |
| 2023 | 33.30% | 37.17% |  |
| ***School Type*** |  |  | **<.001** |
| Middle School | 49.21% | 50.75% |  |
| High School | 50.37% | 49.24% |  |
| ***Sex*** |  |  | **<.001** |
| Male | 50.37% | 55.78% |  |
| Female | 49.21% | 44.21% |  |
| ***Race/Ethnicity*** |  |  | **<.001** |
| White | 53.19% | 40.84% |  |
| Black | 12.33% | 18.03% |  |
| Hispanic | 25.41% | 35.85% |  |
| Asian | 5.91% | 3.71% |  |
| Other | 1.25% | 1.55% |  |
| ***Sexual Orientation*** |  |  | **<.001** |
| Heterosexual | 69.07% | 66.55% |  |
| Sexual Minority | 14.28% | 13.07% |  |
| Not sure | 13.37% | 20.37% |  |
| ***Tobacco Use in Household*** |  |  | **<.001** |
| Yes | 29.32% | 34.97% |  |
| No | 67.80% | 65.02% |  |
| ***Social Media Usage*** |  |  | **<.001** |
| Never | 8.31% | 8.45% |  |
| Few times a week | 9.29% | 12.05% |  |
| 1-2 hours a day | 25.75% | 24.20% |  |
| 3+ hours a day | 56.20% | 55.33% |  |
| ***Average Grades*** |  |  | **<.001** |
| Mostly A-Bs | 73.49% | 66.05% |  |
| Mosty C-Ds | 15.81% | 17.51% |  |
| Mostly Fs | 2.43% | 3.55% |  |
| No Grade/Not sure | 7.58% | 12.88% |  |
| Note: Boldface indicates statistical significance | | | |
