# Supplementary material for: Mental health outcomes associated with electronic cigarette use, combustible tobacco use, and dual use among U.S. adolescents: Insights from the National Youth Tobacco Survey
Source: PLOS Ment Health. 2025 Jul 23;2(7):e0000370. doi: 10.1371/journal.pmen.0000370 (PMC12798231; doi:10.1371/journal.pmen.0000370)
Supplement: S3 Table — (DOCX) [file pmen.0000370.s003.docx]

| **S3 Table:** Unadjusted and Adjusted Odds Ratios for Association between Independent Variables and Depression | | |
| --- | --- | --- |
| **Variable:** | **Unadjusted OR (95% CI)** | **Adjusted OR (95% CI)** |
| ***Smoking Status*** |  |  |
| CTP-Only Use | **1.87 (1.62, 2.16)** | ***1.52 (1.29,1.78)*** |
| E-cigarette Only Use | **2.06 (1.86,2.29)** | **1.67 (1.50,1.85)** |
| Dual Use | **2.58 (2.34,2.86)** | **1.90 (1.69, 2.14)** |
| Non-Use | *1* | *1* |
| ***School Type*** |  |  |
| Middle School | **1.17 (1.10, 1.25)** | 1.02 (0.95,1.09) |
| High School | *1* | *1* |
| ***Sex*** |  |  |
| Female | **1.91 (1.79,2.03)** | **1.54 (1.43,1.65)** |
| Male | *1* | *1* |
| ***Race/Ethnicity*** |  |  |
| White | *1* | *1* |
| Black | **1.15 (1.05,1.26)** | **1.14 (1.02,1.26)** |
| Hispanic | **1.15(1.07,1.23)** | **1.11 (1.03,1.20)** |
| Asian | 1.47 (0.97,1.37) | **1.38 (1.18,1.62)** |
| Other | 1.05 (0.83, 1.33) | 1.12 (0.87,1.42) |
| ***Sexual Orientation*** |  |  |
| Heterosexual | *1* | *1* |
| Gay, Lesbian, Bisexual | **4.11 (3.80,4.44)** | **3.06 (2.81,3.34)** |
| Not sure | **1.54 (1.41,1.69)** | **1.61 (1.46,1.77)** |
| ***Tobacco use in Household*** |  |  |
| Yes | **1.83 (1.71,1.95)** | **1.45 (1.35,1.56)** |
| No | *1* | *1* |
| ***Social Media Usage*** |  |  |
| Never | *1* | *1* |
| Few times a week | **1.19 (1.01,1.40)** | 1.01 (0.85, 1.21) |
| 1-2 hours a day | 1.07 (0.93, 1.23) | 1.04 (0.89, 1.22) |
| 3+ hours a day | **1.92 (1.69,2.19)** | **1.48 (1.28,1.71)** |
| ***Average Grades*** |  |  |
| Mostly A-Bs | *1* | *1* |
| Mostly C-Ds | **1.79 (1.65, 1.94)** | **1.56 (1.43, 1.71)** |
| Mostly Fs | **4.14 (3.51,4.88)** | **3.31 (2.71, 4.04)** |
| No Grade/Not sure | 1.12 (1.00,1.25) | 1.08 (0.95, 1.23) |
| Note: Boldface indicates statistical significance. | | |
