# Supplementary material for: Mental health outcomes associated with electronic cigarette use, combustible tobacco use, and dual use among U.S. adolescents: Insights from the National Youth Tobacco Survey
Source: PLOS Ment Health. 2025 Jul 23;2(7):e0000370. doi: 10.1371/journal.pmen.0000370 (PMC12798231; doi:10.1371/journal.pmen.0000370)
Supplement: S4 Table — (DOCX) [file pmen.0000370.s004.docx]

| **S4 Table:** Unadjusted and Adjusted Odds Ratios for Association between Independent Variables and Anxiety | | |
| --- | --- | --- |
| **Variable:** | **Unadjusted OR (95% CI)** | **Adjusted OR (95% CI)** |
| ***Smoking Status*** |  |  |
| CTP-Only Use | **1.61 (1.40, 1.86)** | **1.39 (1.18, 1.63)** |
| E-cigarette-Only Use | **1.91 (1.73, 2.11)** | **1.48 (1.34, 1.65)** |
| Dual Use | **2.19 (1.98, 2.45)** | **1.58 (1.40, 1.78)** |
| Non-Use | 1 | 1 |
| ***School Type*** |  |  |
| Middle School | **1.16 (1.09, 1.23)** | 0.94 (0.87,1.01) |
| High School | 1 | 1 |
| ***Sex*** |  |  |
| Female | **2.81 (2.64, 2.99)** | **2.32 (2.17, 2.48)** |
| Male | 1 | 1 |
| ***Race/Ethnicity*** |  |  |
| White | 1 | 1 |
| Black | **0.88 (0.80, 0.96)** | **0.83 (0.75, 0.92)** |
| Hispanic | 0.96 (0.90, 1.03) | 0.93 (0.87, 1.01) |
| Asian | 0.94 (0.79, 1.11) | 1.09 (0.93, 1.28) |
| Other | 0.91 (0.73, 1.15) | 1.05 (0.83, 1.32) |
| ***Sexual Orientation*** |  |  |
| Heterosexual | 1 | 1 |
| Gay, Lesbian, Bisexual | **4.46 (4.13, 4.81)** | **3.10 (2.84, 3.37)** |
| Not sure | **1.58 (1.45, 1.72)** | **1.61 (1.46, 1.78)** |
| ***Tobacco use in Household*** |  |  |
| Yes | **1.78 (1.67, 1.90)** | **1.39 (1.29,1.50)** |
| No | 1 | 1 |
| ***Social Media Usage*** |  |  |
| Never | 1 | 1 |
| Few times a week | **1.19 (1.02, 1.40)** | 1.02 (0.86, 1.22) |
| 1-2 hours a day | **1.25 (1.10, 1.43)** | **1.21 (1.04, 1.40)** |
| 3+ hours a day | **2.09 (1.85, 2.37)** | **1.57 (1.37, 1.81)** |
| ***Average Grades*** |  |  |
| Mostly A-Bs | 1 | 1 |
| Mostly C-Ds | **1.41 (1.30, 1.52)** | **1.30 (1.19, 1.42)** |
| Mostly Fs | **2.75 (2.34, 3.24)** | **2.30 (1.88, 2.82)** |
| No Grade/Not sure | 0.90 (0.80, 1.01) | 0.93 (0.82, 1.06) |
| Note: Boldface indicates statistical significance. | | |
