# Supplementary material for: Mental health outcomes associated with electronic cigarette use, combustible tobacco use, and dual use among U.S. adolescents: Insights from the National Youth Tobacco Survey
Source: PLOS Ment Health. 2025 Jul 23;2(7):e0000370. doi: 10.1371/journal.pmen.0000370 (PMC12798231; doi:10.1371/journal.pmen.0000370)
Supplement: S5 Table — (DOCX) [file pmen.0000370.s005.docx]

| **S5 Table:** Unadjusted and Adjusted Odds Ratios for Association between Independent Variables and Psychological Distress | | |
| --- | --- | --- |
| **Variable:** | **Unadjusted OR (95% CI)** | **Adjusted OR (95% CI)** |
| ***Smoking Status*** |  |  |
| CTP-Only Use | **1.70 (1.49, 1.94)** | **1.44 (1.25, 1.65)** |
| E-cigarette-Only Use | **2.13 (1.94, 2.33)** | **1.63 (1.50, 1.78)** |
| Dual Use | **2.48 (2.27, 2.71)** | **1.76 (1.59, 1.94)** |
| Non-Use | 1 | 1 |
| ***School Type*** |  |  |
| Middle School | **0.79 (0.75, 0.83)** | **0.91 (0.86, 0.96)** |
| High School | 1 | 1 |
| ***Sex*** |  |  |
| Female | **2.49 (2.36, 2.63)** | **2.04 (1.93, 2.16)** |
| Male | 1 | 1 |
| ***Race/Ethnicity*** |  |  |
| White | 1 | 1 |
| Black | 0.96 (0.88, 1.03) | 0.92 (0.84, 1.01) |
| Hispanic | 1.06 (1.00, 1.12) | 1.03 (0.97, 1.10) |
| Asian | 1.16 (1.00, 1.33) | **1.39 (1.23, 1.57)** |
| Other | 0.87 (0.69, 1.08) | 0.99 (0.79, 1.25) |
| ***Sexual Orientation*** |  |  |
| Heterosexual | 1 | 1 |
| Gay, Lesbian, Bisexual | **4.72 (4.41, 5.05)** | **3.30 (3.06, 3.55)** |
| Not sure | **1.48 (1.36, 1.60)** | **1.56 (1.43, 1.70)** |
| ***Tobacco use in Household*** |  |  |
| Yes | **1.88 (1.78, 1.99)** | **1.52 (1.43, 1.61)** |
| No | 1 | 1 |
| ***Social Media Usage*** |  |  |
| Never | 1 | 1 |
| Few times a week | **1.43 (1.25, 1.68)** | **1.22 (1.05, 1.41)** |
| 1-2 hours a day | **1.44 (1.29, 1.62)** | **1.35 (1.19, 1.54)** |
| 3+ hours a day | **2.46 (2.20, 2.74)** | **1.81 (1.60, 2.05)** |
| ***Average Grades*** |  |  |
| Mostly A-Bs | 1 | 1 |
| Mostly C-Ds | **1.57 (1.46, 1.68)** | **1.42 (1.31, 1.53)** |
| Mostly Fs | **3.27 (2.77, 3.86)** | **2.58 (2.13, 3.12)** |
| No Grade/Not sure | 0.91 (0.82, 1.01) | 0.93 (0.85, 1.03) |
| Note: Boldface indicates statistical significance. | | |
